# Recent Advances in Well-Designed Therapeutic Nanosystems for the Pancreatic Ductal Adenocarcinoma Treatment Dilemma

**DOI:** 10.3390/molecules28031506

**Published:** 2023-02-03

**Authors:** Xiao-Yan Yang, Yuan-Fei Lu, Jian-Xia Xu, Yong-Zhong Du, Ri-Sheng Yu

**Affiliations:** 1 Department of Radiology, Second Affiliated Hospital, School of Medicine, Zhejiang University, 88 Jiefang Road, Hangzhou 310009, China; 2 Department of Radiology, The Second Affiliated Hospital of Zhejiang Chinese Medical University, 318 Chaowang Road, Hangzhou 310005, China; 3 Institute of Pharmaceutics, College of Pharmaceutical Sciences, Zhejiang University, 866 Yuhangtang Road, Hangzhou 310058, China

**Keywords:** pancreatic ductal adenocarcinoma, nanomedicine, cancer therapy, nanomaterials, drug delivery

## Abstract

Pancreatic ductal adenocarcinoma (PDAC) is a highly malignant tumor with an extremely poor prognosis and low survival rate. Due to its inconspicuous symptoms, PDAC is difficult to diagnose early. Most patients are diagnosed in the middle and late stages, losing the opportunity for surgery. Chemotherapy is the main treatment in clinical practice and improves the survival of patients to some extent. However, the improved prognosis is associated with higher side effects, and the overall prognosis is far from satisfactory. In addition to resistance to chemotherapy, PDAC is significantly resistant to targeted therapy and immunotherapy. The failure of multiple treatment modalities indicates great dilemmas in treating PDAC, including high molecular heterogeneity, high drug resistance, an immunosuppressive microenvironment, and a dense matrix. Nanomedicine shows great potential to overcome the therapeutic barriers of PDAC. Through the careful design and rational modification of nanomaterials, multifunctional intelligent nanosystems can be obtained. These nanosystems can adapt to the environment’s needs and compensate for conventional treatments’ shortcomings. This review is focused on recent advances in the use of well-designed nanosystems in different therapeutic modalities to overcome the PDAC treatment dilemma, including a variety of novel therapeutic modalities. Finally, these nanosystems’ bottlenecks in treating PDAC and the prospect of future clinical translation are briefly discussed.

## 1. Introduction

### 1.1. Current Status of and Dilemmas in PDAC Treatment

Pancreatic cancer is one of the most aggressive and lethal solid tumors worldwide and is the third leading cause of death in men and women combined, with a very low five-year overall survival rate of 11% [1]. Although the five-year survival rate (9%) [2,3] for pancreatic cancer has increased slightly in the past few years, its incidence has continued to increase by about 1% annually [1], and it is predicted to be the second leading cause of cancer death in the United States by 2030 [4]. Due to the aggressive nature of pancreatic cancer and the difficulty of early diagnosis, the number of deaths and cases is almost equal. According to a report, 496,000 cases of pancreatic cancer were diagnosed worldwide in 2020, including 466,000 deaths [5]. Pancreatic cancers are generally divided into endocrine and exocrine cancers, among which pancreatic ductal adenocarcinoma (PDAC) is an exocrine tumor, accounting for the majority (90%) of all pancreatic cancers [6,7]. PDAC rarely shows clinical symptoms until it develops into the advanced stage, resulting in a delay in diagnosis and treatment [8].

The current clinical treatment for PDAC mainly relies on surgery and chemotherapy. For resectable PDAC, surgery is the first choice, followed by adjuvant chemotherapy. Although surgical treatment improves the five-year survival rate, only 15–20% of cases qualify for surgical resection and the recurrence rate after surgery is as high as 85% [9]. The standard first-line chemotherapy regimen for patients with advanced PDAC is gemcitabine (GEM) plus albumin-bound (nab) paclitaxel or FOLFIRONOX/modified FOLFIRONOX (a combination of 5-fluorouracil (5-FU), leucovorin, irinotecan, and oxaliplatin) [10,11]. Nab-paclitaxel plus GEM had a significant survival benefit compared with GEM alone (8.5 months vs. 6.7 months). The FOLFIRINOX regimen further improved the median survival to 11.1 months [12]. Although these chemotherapy regimens represent a major breakthrough in the treatment of PDAC, they have been associated with more severe side effects. Moreover, although chemotherapy does improve the survival of patients to some extent, it is far from satisfactory, and the overall prognosis of PDAC is still poor. In addition to chemotherapy, researchers are eager to improve outcomes using other therapies, such as radiotherapy, targeted therapy, and immunotherapy. Unfortunately, the results have been disappointing. These therapies, which have been successful in many other types of tumor, have yet to make breakthroughs in PDAC [13].

The failure of multiple treatments indicates that there are complex dilemmas and challenges in the treatment of PDAC. In fact, PDAC is a very unique malignancy. The characteristics of PDAC, such as high molecular heterogeneity [14], drug resistance [15], an immunosuppressive microenvironment [16], a highly fibrotic dense matrix [16], and high hypoxia [17], pose great difficulties in its treatment. High molecular heterogeneity is one of the dilemmas in the treatment of PDAC, and gives PDAC obvious invasive and metastatic ability [14]. Drug resistance is another major dilemma in the treatment of PDAC which makes it difficult for chemotherapy drugs and targeted therapies to play a role [15]. The immunosuppressive microenvironment makes it difficult for traditional immunotherapy to achieve satisfactory results in PDAC [16]. The highly fibrotic dense matrix is another huge dilemma in the treatment of PDAC; it is not only related to tumor progression and metastasis, but also forms a tight barrier for drug penetration. In addition, the highly hypoxic microenvironment, as another major dilemma, has a significant impact on tumor invasiveness, metabolism, and treatment resistance [17]. Our understanding of the complex molecular composition and specific microenvironment of PDAC has made great progress, and the key is to overcome these dilemmas in a targeted way. Therefore, novel experimental designs and well-designed therapeutic approaches that specifically address this challenge are needed to achieve effective treatment.

### 1.2. Well-Designed Therapeutic Nanosystems for PDAC

**Figure 1:**
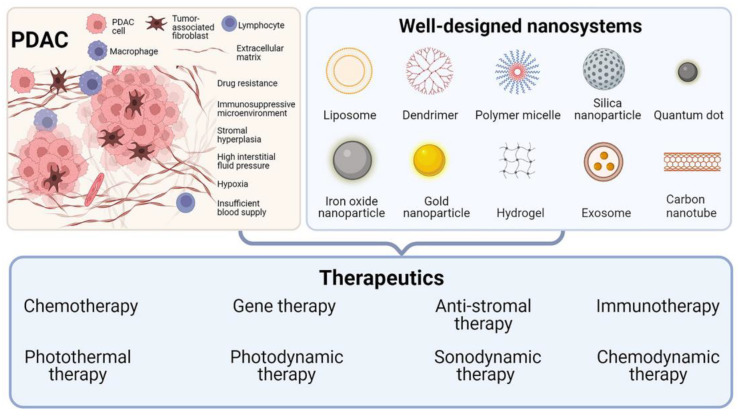
Well-designed nanosystems for different PDAC therapeutics. Created using BioRender.com.

In recent years, the application of nanotechnology in cancer research has received increasing attention. Nanomaterials show great potential in drug delivery, imaging, and cancer treatment due to their unique properties [18,19,20]. Compared with traditional drugs, nanomedicines have many advantages, such as passive targeting of tumor tissue through the enhanced permeation and retention (EPR) effect, improved circulation, high drug loading capacity, and even intrinsic diagnostic and therapeutic properties [21]. In 2013, nab-paclitaxel plus GEM was reported to improve overall survival and progression-free survival in patients with metastatic PDAC, and was approved for first-line treatment in patients with advanced PDAC [22]. After years of clinical application, nab-paclitaxel has been proven to improve the prognosis of patients with PDAC effectively. In recent years, an increasing number of nanomedicines for PDAC treatment have been used in clinical trials (Table 1), which indicates that nanomedicine has great transformation potential in PDAC treatment. In addition to traditional nanomaterials (such as micelles and liposomes), new functional nanomaterials are also being developed, such as lanthanide nanoparticles (NPs), superparamagnetic iron oxide (SPIO) NPs, gold (Au) NPs, quantum dots (QDs), carbon nanotubes (CNTs), mesoporous silica NPs (MSNPs), metal–organic framework (MOF)-based materials, biomimetic nanomaterials, etc., which can incorporate imaging agents and/or therapeutic drugs [23]. The superior properties of these nanomaterials allow them to be refined and engineered, resulting in intelligent and well-designed nanosystems that integrate multiple functions. This review does not just refer to NPs for the treatment of PDAC. What we call a nanosystem is a multifunctional system based on a variety of nanomaterials such as NPs. NPs are the most basic and important components of nanosystems. There are other components, including a modification component, a drug component, a targeting component, a stimulus-response component, etc., which ultimately constitute a well-designed nanosystem. This well-designed nanosystem can specifically target defects in tumors to achieve a precision treatment effect. Through the rational design of nanosystems, researchers have constructed nanosystems that can overcome PDAC drug resistance, penetrate PDAC matrix barriers, reverse PDAC immune suppression, etc. In addition to conventional chemotherapy, those well-designed nanosystems show great potential for use in various novel cancer treatment modalities. This review aims to summarize the current state of the art in well-designed nanosystems for PDAC therapy. We emphasize the specificity of these nanosystems in overcoming the therapeutic dilemma of PDAC. This review will discuss the logical design and functionality of these nanosystems in terms of different therapeutic modalities, including chemotherapy, gene therapy, immunotherapy, anti-stroma therapy, photothermal therapy (PTT), photodynamic therapy (PDT), sonodynamic therapy (SDT), and chemodynamic therapy (CDT) (Figure 1). Finally, the bottleneck of these nanotechnologies in PDAC treatment and the prospect of clinical translation in the future will be briefly discussed. Through an in-depth summary and analysis, this review aims to provide cutting-edge ideas for further research on strategies to overcome the dilemma of PDAC treatment based on well-designed nanosystems.

## 2. Well-Designed Nanosystems for Different PDAC Therapeutics

### 2.1. Nanosystems Designed for PDAC Chemotherapy

A variety of chemotherapy drugs, including GEM, paclitaxel, irinotecan, fluorouracil, oxaliplatin, capecitabine, doxorubicin, and curcumin, are used in the treatment of PDAC. However, the efficacy of the drugs is limited by non-specific distribution, drug metabolism, and intrinsic toxicity [24]. To address these issues, a promising option is to implement nanomaterial-based therapeutics in PDAC. Table 2 summarized recent studies on the delivery of PDAC chemotherapy drugs based on nanodelivery systems. GEM is considered the gold standard and is the first FDA-approved agent as monotherapy for advanced PDAC. Despite initial clinical success as a first-line treatment for PDAC, the therapeutic efficacy of GEM is limited by rapid metabolism, high-dose toxicity, and drug resistance [25]. GEM is easily metabolized to inactive 2′, 2′-difluorodeoxyuridine by deoxycytidine deaminase present in the plasma and liver, thus resulting in a very short plasma half-life of 15–20 min. To overcome these limitations and improve safety, a variety of GEM delivery systems have been developed. In addition to traditional liposomes, micelles, and polymer NPs, many novel nanomaterials such as MOF, QDs, hydrogels, and exosomes have also been developed (Table 2). Loading GEM in nanocarriers improves its pharmacokinetic parameters and facilitates entry into target cells via passive or active targeting, thus improving drug availability and reducing high-dose toxicity. Li et al. reported an autologous exosome (ExoGEM) as a drug carrier for targeted delivery of GEM for the treatment of PDAC [26]. ExoGEM showed superior therapeutic efficacy against PDAC, extending survival in a dose-responsive manner with minimal damage to normal tissue. Various stimulus-responsive (such as pH, enzymes, temperature, redox reactions, magnetic fields, light, ultrasound, etc.) nanodelivery systems have been developed [27]. Based on the properties of the tumor microenvironment (TME) or external stimuli, nanosystems can release active therapeutic components at specific sites in a controlled and targeted manner. Strategies for size or morphology transformation in response to stimuli have emerged in recent years, which can make the best use of nanosystems in different situations. Sheng et al. designed a GEM-loaded matrix metalloproteinase 2 (MMP-2)-responsive transformable beaded nanofibril, which integrates the merits of nanofibril and small-sized NPs [28]. In general, the longer the length of the fibril, the longer the circulation time, but the lower the penetration and internalization. Instead, small-size NPs with positive charges are the best choice for penetration and internalization. This nanosystem can self-assemble into negatively charged beaded nanofibrils with prolonged circulation time under physiological conditions. However, upon reaching the tumor tissue, in response to MMP-2, the nanofibrils transform into positively charged small particles, which can be effectively internalized by tumor cells. This fiber-to-sphere transition strategy takes full advantage of nanomaterials with different sizes and morphologies, allowing GEM to overcome multiple obstacles to be internalized into cells.

Due to its hydrophilic nature, it is difficult for GEM to enter cells via passive diffusion and it relies on human nucleoside transporters (hNTs) instead. Human NT is a family of membrane proteins containing a variety of protein subtypes, among which human equilibrative nucleoside transporter 1 (hENT1) mediates most GEM transport. Therefore, hENT1 deficiency is one of the most important reasons for cell resistance to GEM. To overcome GEM resistance induced by low hENT1 expression, Guo et al. reported a GEM-loaded human serum albumin NP (GEM-HSA-NP) which could be taken into tumor cells through endocytosis, thus bypassing the transport of hENT1 [29]. In vivo and in vitro studies showed that GEM-HSA-NP was more effective than GEM in inhibiting tumor growth regardless of whether hENT1 expression level was high or low. Another study developed hyaluronic acid-functionalized pH-sensitive liposomes (HA-pSL) to investigate whether intracellular delivery could overcome GEM resistance [30]. They hypothesized that HA-pSL would be internalized through CD44-mediated cellular endocytosis. The outcomes demonstrated that the nanosystem showed increased cell uptake and in vitro cytotoxicity, but did not eliminate resistant GR2000 cells. In vivo efficacy studies showed that the nanosystem only slightly enhanced the efficacy of GEM therapy, and the tumor was not eliminated. Their results suggest that not all cases are simply “transport-based resistance”. Before designing better nanosystems, the properties of nanomaterials and the complex biological interactions with tumor cells and TME need to be fully understood.

Although FOLFIRINOX has a better response rate than GEM, the side effects associated with the treatment are also more severe. Irinotecan, a component of the FOLFIRINOX regimen, significantly affects this toxic response. Although MM-398 (nanoliposomal irinotecan) has been approved by the FDA for the treatment of patients with PDAC who do not respond to GEM therapy, its gastrointestinal and bone marrow toxicity effects remain relatively high. To address the possible failure of liposomal carriers to improve the safety of highly toxic drugs such as irinotecan, Liu et al. developed lipid bilayer-coated mesoporous silica NPs (LB-MSNPs) for high-dose irinotecan loading [31]. The stability and loading capacity of the nanocarrier improved the biodistribution, circulating half-life, and drug tumor content of irinotecan compared with the MM-398 liposome, resulting in a more effective anticancer effect and lower toxicity in a robust treatment-resistant KRAS-induced pancreatic cancer (KPC) model.

In addition to single-drug delivery, a variety of co-delivery vehicles are also being developed. For instance, Shabana et al. designed a thermosensitive and biodegradable hydrogel encapsulating targeted NPs for the local and sustained co-delivery of GEM and paclitaxel in PDAC [32]. Chen et al. constructed a pH-activatable core-shell nanobomb for the co-delivery of autophagy inhibitor and GEM [33]. Lei et al. developed polymeric micelles bearing active formats of irinotecan and oxaliplatin [34]. These findings suggested that combined delivery, enabled by nanotechnology, may further improve the feasibility of multidrug treatment for advanced PDAC.

Collectively, the main difficulties in PDAC chemotherapy are poor bioavailability, drug resistance, and toxic side effects. Therefore, the main design concepts of nanosystems in PDAC chemotherapy are to improve drug bioavailability, overcome drug tolerance, and reduce drug side effects. To this end, researchers have designed a series of drug delivery nanosystems, including a ligand-targeted delivery system, a stimulus-response delivery system, a biomimetic delivery system, a collaborative treatment system, and a cascade-targeted delivery system. Recently, preclinical research has been devoted to developing novel nanocarriers. However, the nanocarriers that have entered clinical application mainly focus on liposomes. Although the development of new nanomaterials and vectors is a promising area, perhaps researchers should pay more attention to further studying the performance of existing vectors to ensure higher clinical translational potential.

**Table 2:** Recent advances in chemotherapy drug delivery nanosystems for PDAC.

Chemotherapy Drugs	Nanosystems	Animal model	Ref
GEM	GEM-conjugated polymeric micelle	MIA PaCa-2 orthotopic model	[35]
GEM	MOF NPs	PANC-1 cells	[36]
GEM	GEM-loaded albumin NPs	PDAC patient-derived xenograft (PDX) subcutaneous model	[29]
GEM	Self-healing pH- and enzyme stimulus-responsive hydrogels	AsPC-1 subcutaneous model	[37]
GEM	Autologous exosomes	PANC-1 subcutaneous model	[26]
GEM	Plectin-1-targeted AuNPs	PANC-1 orthotopic model	[38]
GEM	Transferrin (Tf)-conjugated polymer-coated MSNPs	MIA PaCa-2 cells	[39]
GEM	Dual enzymatic reaction-assisted CdSe/ZnS QDs	BxPC-3 subcutaneous model	[40]
GEM and paclitaxel	Lipid-coated MSNPs	PANC-1 subcutaneous model	[41]
GEM and paclitaxel	Thermosensitive and biodegradable hydrogel encapsulating targeted NPs	PANC-1 cells	[32]
GEM and doxorubicin	Protein–gold cluster-capped MSNPs	MIA PaCa-2 subcutaneous model	[42]
GEM and doxorubicin	Photo- and thermoresponsive multicompartment hydrogels	-	[43]
Irinotecan	Lipid bilayer-coated MSNPs	KPC-derived orthotopic model	[31]
Curcumin	Tf-targeted PEGylated curcumin-loaded MSNPs	MIA PaCa-2 subcutaneous model	[44]
Curcumin	SPIO NPs of curcumin	HPAF-II orthotopic model	[45]

### 2.2. Gene Therapy through Tailored Nanosystems

Most instances of PDAC arise from a precursor lesion called pancreatic intraepithelial neoplasia (PanIN), which progresses by acquiring genetic alterations and eventually develops into overt PDAC [8]. Multiple genetic alterations are present in PDAC, the most common of which is the activation of the *KRAS* proto-oncogene, which is present in more than 95% of human PDAC [46,47,48]. Oncogenic *KRAS* may be the main driving force behind PDAC, which was identified in PanIN [47]. Several other common gene mutations included *TP53*, *SMAD4*, and *CDKN2A*, each of which was mutated in >50% of different but mostly overlapping patients [49,50]. Mutations in genes with an incidence of 5–10% have also been identified, including *KDM6A*, *BCORL1*, *RBM10*, *MLL3* (also known as *KMT2C*), *ARID1A*, and *TGFBR2* [51,52,53]. The pathogenesis of PDAC is the continuous accumulation of somatic mutations in oncogenes and tumor suppressor genes. Therefore, gene therapy is a promising approach to PDAC treatment. Gene therapy, defined as the deletion, insertion, modification, or addition of genes in target cells, has shown significant therapeutic effects in recent preclinical and clinical studies [54]. Therapeutic genes include plasmid DNA (pDNA), mRNA, microRNA (miRNA), short interfering RNA (siRNA), and antisense oligonucleotides [54]. Their effects are limited by poor stability in vivo, immune system activation, insufficient tumor delivery, and barriers to transport across the plasma membrane to the cytoplasm [55]. Vectors used to deliver genes include viral vectors and non-viral vectors. Viral vectors have shown high transduction efficiency. However, their potential carcinogenicity, immunogenicity, and high production cost limit their application [54]. Therefore, non-viral vectors have received a great deal of attention. In addition to traditional cationic liposomes and polymers, a variety of emerging nanomaterials such as gold nanoclusters, layered double hydroxides (LDH), CNTs, and graphene oxide (GO) have been developed for gene delivery (Table 3).

*KRAS^G12D^* has a mutation rate of more than 90% in PDAC, which promotes tumorigenesis, development, and metastasis [56]. Therefore, *KRAS* mutation inhibition holds great promise for effective gene therapy strategies for PDAC. Unfortunately, traditional gene therapy faces many huge biological and technical challenges. Yu et al. used a synthetic nucleic acid analog called peptide nucleic acid (PNA) [56]. Compared with conventional negatively charged DNA or RNA, neutral PNA is more stable in recognizing and forming PNA-DNA hybrids with target mutant sequences. LDH nanosheets have attracted great interest due to their good stability and dispersion and high gene delivery efficiency. Therefore, they designed a PNA-based gene therapy strategy. LDH is used as a PNA carrier to effectively deliver PNA to PDAC cells and achieve mutation silencing of *KRAS*. Through the proton sponge effect of LDH, PNA is able to be released from lysosomes into the nucleus after being phagocytosed by the cell, thus having a significant therapeutic effect on PDAC [56]. In addition, to overcome the instability and low absorption efficiency of siRNA in vivo, Yin et al. used functionalized GO nanosheets as gene carriers to co-deliver *HDAC1* and *KRAS* siRNA. Double silencing of *HDAC1* and *KRAS* significantly inhibited cell proliferation and tumor growth [70].

Traditional cationic polymeric gene vectors are closely connected to negatively charged DNA through electrostatic interaction, which is difficult to dissociate after reaching cells, resulting in low transfection efficiency. To this end, Zhang et al. constructed a reactive oxygen species (ROS)-responsive gene delivery vector by encapsulating the polymer B-PDEAEA and DNA complex in IR780-loaded liposomes [67]. IR780 in the nanosystem can produce a large number of ROS under ultrasound irradiation, triggering the charge reversal of the redox-responsive polymer, thus releasing *TRAIL* pDNA [67]. *TRAIL* is a therapeutic gene that specifically eliminates tumor cells without damaging normal cells. Additionally, the modification of polyethylene glycol (PEG) can further maintain the stability of therapeutic genes and prolong blood circulation time, thus improving the efficiency of gene transfection. Most importantly, a combination of this gene delivery system and ultrasound can achieve superior biocompatibility and therapeutic effect in vitro and in vivo.

In addition to targeting PDAC tumor cells, Jiang et al. designed a gene delivery strategy targeting pancreatic stellate cells (PSCs) [62]. Due to the large number of PSCs present in the PDAC microenvironment and located near blood vessels, targeting PSC helps overcome the difficulty of delivering genes to PDAC tumor cells [62]. In their study, branched polyethyleneimine was used to deliver the *TRAIL* gene to PSC and induce PSC to produce TRAIL protein, which was non-toxic to PSC but could induce the death of neighboring PDAC cells. In conclusion, they demonstrate that PSC, as a major stromal component in the PDAC microenvironment, is an ideal host for gene therapy strategies and in developing novel modalities for gene therapy for PDAC.

In addition to the *KRAS* mutations observed in 90% of patients with PDAC, mutations in the tumor suppressor gene *P53* are also observed in 50–80% of patients. Moreover, about 55% of patients have both *KRAS* and *P53* mutations, which lead to an increase in glucose metabolites, including deoxycytidine triphosphate (dCTP) [58]. dCTP can be inserted into DNA strands for replication and will compete with the active metabolite of GEM in the nucleus, thus making PDAC resistant to GEM. In this case, overcoming drug resistance by simultaneously inhibiting the *KRAS* and *P53* genes can lead to effective PDAC therapy. The clustered regularly interspaced short palindromic repeats-associated protein 9 (CRISPR-Cas9) system is an emerging gene editing therapy [71]. The adenine base editor (ABE) is a more advanced gene editing strategy [72]. Won et al. constructed a Cas9 and ABE-based ribonucleoprotein gene editor that utilizes a biocompatible liposome as a delivery nanosystem for the simultaneous *KRAS* and *P53* gene editing of PDAC [58]. Multiple biological analyses of in vitro and in vivo models verified the effective killing of PDAC cancer cells after co-treatment with double gene editing and GEM [58]. Overall, they provide a combined therapeutic strategy utilizing gene editing systems and may be a promising approach for the treatment of drug-resistant PDAC.

In conclusion, nanosystem-based gene therapy has great potential in PDAC treatment because it can prevent DNA/RNA degradation and enhance in vivo stability and gene transfection efficiency. A large number of preclinical studies have shown that gene therapy may bring new hope for PDAC treatment. However, developing safe and efficient vectors is extremely important to achieve effective treatment. The development of a variety of novel nano-gene delivery systems may uncover a new solution to PDAC treatment in the future. Furthermore, considering the heterogeneity of PDAC tumors and the complexity of the TME, single-target gene therapy may have limited efficacy. Therefore, multi-mutation gene therapy or a combination of multiple therapeutic modalities may achieve a better synergistic therapeutic effect.

### 2.3. Nanosystems Designed to Overcome PDAC Stroma

An important reason for the poor prognosis of PDAC is its specific TME, which includes a highly fibrotic tumor stroma. The stromal component of PDAC is up to 90% of the entire tumor [12]. This specific TME is a highly dynamic system composed of multiple cellular components and an extracellular matrix (ECM). The cellular components include PSCs, cancer-associated fibroblasts (CAFs), immune cells, and endothelial cells. ECM is composed of hyaluronic acid, collagen fiber, fibronectin, and soluble growth factors such as transforming growth factor-β (TGF-β), vascular endothelial growth factor, and fibroblast growth factor [12]. The dense tumor stroma of PDAC results in elevated interstitial fluid pressure, poor blood supply, and hypoxia in the TME. The interaction between stroma and tumor cells has a significant impact on the progression, invasion, and metastasis of PDAC [6]. Additionally, the dense fibrotic stroma severely impedes drug penetration into the PDAC tumor tissue, which leads to poor efficacy of the PDAC drug treatment. Therefore, modulation of the PDAC stroma to facilitate treatment is a highly effective strategy. A series of nanosystem-based matrix modulation strategies have been developed, including targeting PSCs, CAFs, ECM components, matrix-related signal transduction pathways, etc. (Table 4).

In the PDAC stroma, PSCs are the main source of CAFs. Activated PSCs are converted into CAFs, which form a dense ECM network by producing large amounts of fibronectin, collagen, and α smooth muscle actin (α-SMA) [74]. There is increasing evidence that PSCs present in the stroma of PDAC promote the invasion and metastasis of cancer cells by remodeling TME [84]. Therefore, targeting PSCs and remodeling PDAC stroma has proven to be a promising strategy for the treatment of PDAC [85]. Calcipotriol is a ligand for vitamin D receptors, which can inhibit the activation of PSCs and restore them to the resting state [86]. However, its poor water solubility, short plasma half-life, and adverse reactions limit its clinical application. Wang et al. constructed a self-assembled polymer nanosystem for the co-delivery of calcipotriol and the chemotherapy drug SN38 [73]. Their results showed that this nanosystem improved the water solubility of calcipotriol and prolonged the blood circulation time of calcipotriol significantly. After systemic administration, this nanosystem effectively inhibited ECM production, reducing collagen and fibronectin content. This nanosystem effectively promoted the penetration of chemotherapeutic drugs, showing inhibitory effects on primary and metastatic tumors in a PDAC mouse model. In another study, researchers inhibited the activation of PSCs to produce ECM by delivering nitric oxide (NO) [74]. NO is an important biological signaling molecule that can regulate a variety of physiological processes. They used liposomes loaded with a glutathione (GSH)-sensitive NO donor, S-nitroso-N-acetylpenicillamine (SNAP). NO can be rapidly released from SNAP when stimulated by high concentrations of GSH at the tumor site. Next, NO reduced the production of ECM components in PSCs by inhibiting the TGF-β1 pathway and cutting off its downstream pro-fibrotic signal transduction [74]. Due to the reduction in stroma induced by NO, enhanced liposome-GEM intratumor penetration was achieved in the PDAC mouse model. In addition, Feng et al. used polymer NPs coated with CREKA peptide and loaded α-mangostin (CRE-NP(α-M)) to target CAFs and interfere with the TGF-β/Smad signaling pathway to inhibit CAFs from producing ECM [76]. The sequential targeted drug delivery regimen after CRE-NP(α-M) pretreatment showed strong tumor growth inhibition in the orthotopic tumor model.

The TGF-β1 signal transduction pathway not only plays an important role in promoting stroma formation in PDAC [87], but also mediates drug resistance through autocrine or paracrine mechanisms. PDAC tumor cells, or PSCs, contribute to the production of TGF-β1, which can activate PSCs and maintain their myofibroblast phenotype through an autocrine positive feedback mechanism [88]. As a result, the activated PSCs continue to secrete large amounts of ECM components, forming a dense ECM network [89]. Based on the key role of TGF-β1 in the course of forming PDAC stroma, targeting TGF-β1 signal transduction represents a promising approach. Vactosertib (VAC) is a selective small-molecule inhibitor of TGF-β1 receptor kinase. Zhao et al. designed a size-switchable nanoplatform by incorporating paclitaxel into the hydrophobic layer of small nanospheres (NS-TAX), and then, encapsulating them in liposomes loaded with VAC (NS-TAX@Lipo-VAC) [77]. NS-TAX@Lipo-VAC can target the ECM of PDAC and accumulate in the matrix due to its large size. The released NS-TAX is able to penetrate deep into tumors because of its small size. At a later stage, VAC inhibited TGF-β1 signaling in the tumor stroma, leading to a significant decrease in ECM protein, which further promoted the penetration of NS-TAX in tumor tissues. Their nanoplatform effectively reduced ECM production and inhibited tumor progression in fibrosis-rich PDAC animal models. This cascade of penetration strategies combining TGF-β1 signal regulation and size-switchable nanoplatforms has great potential to overcome stroma barriers in PDAC and improve therapeutic outcomes. In another study, Li et al. developed an MMP-2-responsive alternating copolymer carrying LY2109761 and CPI-613 [78]. LY2109761 is a novel TGF-β receptor kinase inhibitor that blocks the interaction between tumor cells and PSCs. CPI-613 is an anti-mitochondrial metabolic agent that can disrupt mitochondrial metabolism in cancer cells. The MMP-2-responsive connector is cleaved in the TME, resulting in dissociation of the nanosystem and the subsequent release of LY2109761 and CPI-613. This strategy significantly inhibited the growth of PDAC tumors by simultaneously blocking stroma production and disrupting mitochondrial metabolism in tumor cells.

**Figure 2:**
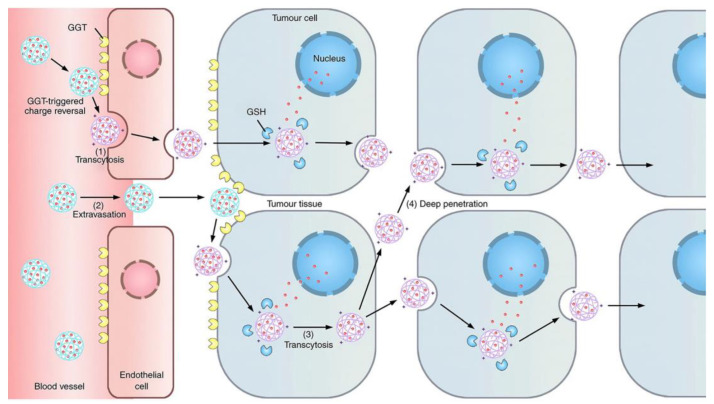
Scheme of the GGT-activatable nanosystem for active tumor penetration through cationization-triggered transcytosis. Reprinted with permission from [79]. Copyright 2019, Springer Nature.

In addition to overcoming the barrier of drug penetration by adjusting the matrix composition, effective deep intratumoral penetration can also be achieved through rational design of the nanosystems. Endothelial cells can actively transport cargo through caveolae-mediated transcytosis to transport it across the capillary wall to the tumor tissue [90]. The cationization of nanocarriers can effectively induce adsorption-mediated transendocytosis and promote the penetration of the carrier through multiple cell layers [91]. Based on this, Zhou et al. designed a γ-glutamyl transpeptidase (GGT)-responsive charge reversal nanosystem [79]. The nanosystem remains neutral or microanionic during blood circulation to ensure effective long circulation. After reaching the tumor site, GGT overexpressed in tumor vascular endothelial cells and on the tumor cell surface triggers the charge reversal of the nanosystem to cationization. This results in rapid caveola-mediated transendocytosis, enabling transendothelial and transcellular transport into the tumor (Figure 2).

In addition to targeting cellular components such as PSCs and CAFs in the stroma, various strategies have been developed to target the ECM. Hyaluronic acid and collagen are the main components of ECM, and several studies have shown that manipulation of these ECM components can effectively alleviate matrix obstruction. For example, ongoing phase III trials of PEGylated recombinant human hyaluronidase in combination with GEM have shown prolonged survival in PDAC treatment [92]. Collagenase has also been used in several studies to degrade collagen in the ECM, reducing interstitial stress and facilitating therapeutic drug penetration [82,83].

To summarize, the stroma composition of PDAC is complex and variable. It is not only related to the progression and metastasis of PDAC, but also affects the immune microenvironment and cell metabolism [93,94]. Anti-stromal therapy holds great promise for the treatment of PDAC, and it can help overcome the drug penetration dilemma of PDAC therapy to promote efficacy. A series of nanosystems have been designed to eliminate the dense stroma of PDAC, including targeting PSCs, CAFs, ECM components, etc. In addition, the design of transformable nanosystems is also a promising strategy for overcoming stroma obstruction. It is important that the complexity of the PDAC stroma system be fully considered in the design of nanosystems, so as to design a more reasonable and refined stroma regulation strategy.

### 2.4. Immunosuppressive Microenvironment-Regulating Nanosystems

Although immunotherapy has greatly changed the outlook for some tumors, such as melanoma and lung cancer, it has had little success in the treatment of PDAC. PDAC is almost completely unable to use FDA-approved immunotherapies, except in <1% of patients with high microsatellite instability [95]. At present, many clinical trials have attempted to evaluate the effects of immunotherapies on PDAC, including immune checkpoint inhibitors (ICIs), cancer vaccines, adoptive cell transfer, etc., but none have shown satisfactory results [16]. The main reason for the hindered efficacy of immunotherapy in PDAC is its highly inhibitory immune microenvironment. This highly inhibitory immune microenvironment lacks cytotoxic T cell (CTL) infiltration but is rich in immunosuppressive cells including dendritic cells (DCs) and tumor-associated macrophages (TAMs), as well as myeloid-derived suppressor cells (MDSCs) and regulatory T cells (Tregs) [96,97,98,99]. Therefore, PDAC is immunologically referred to as an immune “cold” tumor. Researchers in the field of nanomedicine are working hard on new approaches that could effectively change the dilemma of PDAC immunotherapy and are trying to improve the situation of immunotherapy for PDAC by utilizing the advantages of nanomaterials.

The combination of immunogenic cell death (ICD) inducers and ICIs has shown satisfactory results in preclinical studies in a variety of tumors. Some chemotherapeutic agents, such as doxorubicin and oxaliplatin, can increase the response of CTL to “cold” tumors and reverse them to “hot” tumors by inducing ICD. This immunogenic effect occurs due to the ability of these chemotherapeutic agents to trigger an endoplasmic reticulum (ER) stress response that translocates calreticulin (CRT) to the surface of tumor cells [100]. CRT serves as a signal for antigen-presenting cells (APCs) to phagocytose cancer cells, enabling APCs to present endogenous tumor-associated antigens to naive T cells [101]. Moreover, disintegration of the tumor nucleus leads to the release of high-mobility group box 1 protein, which acts as an adjuvant by binding to Toll-like receptor 4 (TLR4) to APCs. In addition, autophagy and ATP release can also increase APC recruitment and immune effects [102]. ICD promotes antigen uptake and presentation by APC through the release of tumor antigens, damage-associated molecular patterns, and proinflammatory cytokines, and ultimately elicits antigen-specific antitumor immune responses [103]. However, the existence of an immune checkpoint can inhibit the killing effect of CTLs. Therefore, ICIs are an effective means of ensuring the tumor killing function of CTLs. Although some progress has been made in the development of ICD inducers and ICI combinations, several challenges remain in clinical translation. Many studies have focused on improving the efficacy of ICD and ICI combination therapy through nanosystems. Liu et al. used lipid-coated MSNPs loaded with irinotecan to explore the immunogenic effect of irinotecan [104]. The results showed that encapsulated irinotecan caused lysosomal alkalinization and ER stress, inducing immunogenicity and PD-L1 expression. In general, the lack of PD-L1 expression in PDAC tumor cells is an important reason for the poor response to the PD-1/PD-L1 blockade [104]. The silica nanosystem induces a more potent chemoimmunotherapeutic response than either free drugs or liposomal irinotecan. Moreover, by inducing the expression of PD-L1, the combined treatment with anti-PD-1 can significantly inhibit tumor growth and improve the survival rate, and is far better than free drugs plus anti-PD-1. In another study, the authors also used lipid-coated MSNPs to load oxaliplatin [105]. In an orthotopic KPC model, the nanosystem showed superior pharmacokinetic profiles and intratumoral drug delivery to free drugs, while inducing significant ICD effects. In addition, combined treatment with anti-PD-1 antibody further enhanced the effect of chemoimmunotherapy. Overall, improving the efficacy of ICD inducers through nanocarriers and combined treatment with ICI may be an effective strategy to improve the treatment dilemma of PDAC. This combinatorial effect raises the possibility of using the multifunctional properties of the nanosystem to co-produce immunomodulators selected to intervene in a range of impediments in PDAC immunotherapy. For example, Luo et al. used this liposomal silica to load the ICD inducer irinotecan into the pores of MSNPs, further loading the outer lipid layer with TLR agonists capable of activating innate immunity [106]. Although ICD effects can improve the delivery of tumor antigens to APCs, the activation, maturation, and enhanced function of APCs require additional co-stimulation [106]. The experimental results showed that the dual-drug delivery vector produced an effective anti-PDAC immune response, including increased DC activation, an increased number of CTLs, and significant tumor shrinkage. These results not only demonstrate the efficacy of co-delivered drug vehicles, but also expand the scope of chemoimmunotherapy for PDAC. Although most studies have focused on the adaptive immune system, the immune microenvironment of PDAC is rich in a large number of dysfunctional APCs. Based on this, Lorkowski et al. designed highly effective innate immune-stimulating NPs to induce strong activation and expansion of APC in TME [107]. The nanosystem is loaded with two innate immune activators, including the pathway agonist of stimulator of interferon genes and the TLR4 pathway activator. The nanosystem stimulated more significant APC amplification than either agonist alone, resulting in a powerful synergistic antitumor immune effect.

**Figure 3:**
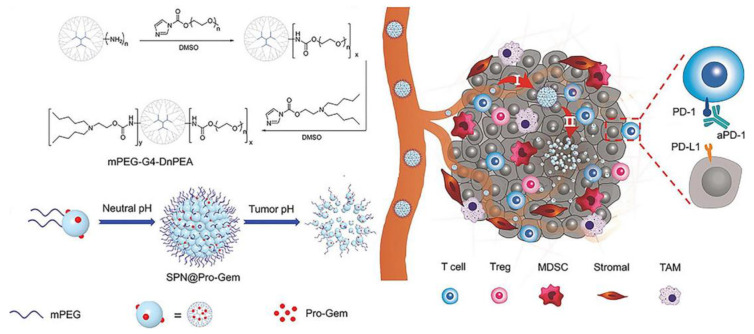
Scheme of the design and mechanism of the pH-sensitive size-transformable tumor-penetrating nanosystem SPN@Pro-Gem. Reprinted with permission from [110]. Copyright 2021, John Wiley and Sons.

In addition to ICD induced by chemotherapeutic agents, other physical mechanisms can also induce ICD while killing tumor cells, such as photothermal and photodynamic effects. Jang et al. constructed a tumor cell-derived exosome loaded with chlorin e6 (Ce6) to target tumor cells and generate ROS in tumor cells after laser irradiation [108]. The ROS produced can not only kill tumor cells, but also further induce anti-tumor immune responses, and eventually, inhibit tumor growth, recurrence, and metastasis. In another study, Yu et al. developed a stimulus-responsive size-transformed liposome in combination with mild hyperthermia and ICI treatment [109]. Mild hyperthermia alleviated tumor hypoxia and increased immune cell infiltration, and combined with ICI therapy, effectively inhibited PDAC tumor growth and metastasis. In addition, the nanosystem can achieve size conversion under temperature stimulation to penetrate deep into tumor tissue and overcome the dense stromal barrier of PDAC. The dense matrix of PDAC is also a major obstacle to the efficacy of immunotherapy; it blocks not only drug penetration but also effector immune cell infiltration. Tong et al. designed a pH-sensitive tumor-penetrating nanosystem that self-assembled into NPs (120 nm) at neutral pH and transformed into small particles (10 nm) at acidic pH (Figure 3) [110]. The released small particles promote the deep penetration of GEM into tumor tissues, induce anti-tumor immune effects, enhance the infiltration of immune cells, and up-regulate the expression level of PD-L1. Ultimately, the combination of anti-PD-1 antibodies with the nanosystem induced robust antitumor immunity.

In addition to immunotherapeutic strategies to activate APCs and enhance the effects of CTLs, TAM is also an important cell in the immunosuppressive microenvironment of PDAC. TAM exhibits two phenotypes, M1-TAM and M2-TAM, in which M2-TAM promotes tumor progression and metastasis and inhibits DCs and CD8^+^T cell function. In contrast, M1-TAM has antitumor effects. Therefore, the polarization of TAM toward the M1 type is an effective antitumor immunotherapy strategy. Zhou et al. developed an exosome-based dual delivery nanosystem for the delivery of oxaliplatin and galectin-9 siRNA [111]. Oxaliplatin not only kills PDAC cells by inhibiting DNA synthesis and repair, but also further induces the ICD effect. Moreover, galectin-9 siRNA polarized M2-TAM by blocking galectin-9/dectin-1. The final results showed that this dual delivery system polarized inhibitory TAM, recruited CTLs, and down-regulated Treg, thus eliciting a potent antitumor immune response. In another study, Parayath et al. constructed M2 peptide-modified polymer NPs for TAM-targeted miR-125b delivery [112]. The results showed that intraperitoneal administration of M2-targeted NPs showed preferential accumulation at the tumor site, and the ratio of M1-to-M2-TAM increased more than fourfold. Due to the poor immunogenicity of PDAC, a single target may not produce a satisfactory therapeutic effect. Li et al. designed an M2-TAM-targeted micelle for simultaneous delivery of the PI3K-γ inhibitor and CSF-1R siRNA [113]. The micelles specifically targeted TAM, effectively reduced the number of M2-TAM, increased the number of M1-TAM, and inhibited the infiltration of MDSCs through dual blockade of the two pathways. This dual inhibitory strategy provided a new approach for the TME remodeling of PDAC and the coordinated activation of antitumor immune effects.

In conclusion, although the low immunogenicity and highly suppressive immune microenvironment of PDAC have largely limited the effectiveness of immunotherapy in PDAC, the development of nanosystems has brought new hope for PDAC immunotherapy. While it is difficult for monotherapy to be effective in PDAC, the nanosystem helps to combine different parts of the immune response pathway to induce effective antitumor immune responses through multiple pathways simultaneously. It is believed that these well-designed nanosystems can achieve new breakthroughs in the field of immunotherapy for PDAC in the future.

### 2.5. Nanosystems Designed for PDAC Photothermal Therapy (PTT)

PTT has become a new and powerful treatment technique because of its non-invasiveness, low damage to normal tissue, and strong therapeutic effect [114,115]. A variety of photothermal agents, such as inorganic (metal NPs, nanosheets, and transition metal oxides) and organic materials (such as polyaniline, cyanine dye, and indocyanine), have been widely developed [116]. Table 5 summarize recent advances in PTT for the treatment of PDAC. Local hyperthermia caused by PTT can increase the blood flow and microvascular permeability of tumor tissue, thus promoting the deep penetration of drugs. Zhao et al. constructed a gold nanoshell-coated mesopore silica nanosystem for combined PTT and chemotherapy treatment of PDAC [117]. The nanosystem provides a cascade-targeting strategy that promotes drug penetration and accumulation through both photothermal and molecular targeting. After inducing PTT at the tumor site, blood perfusion and vascular permeability were improved, which greatly promoted the infiltration and accumulation of nanosystems in tumor tissues. The second step of cascade targeting was to target the TfR on the surface of tumor cells via Tf modification of the nanosystem. Their research demonstrated that the more nanosystems accumulated in the tumor, the better the photothermal warming, which, in turn, promoted the accumulation of more nanosystems, creating a positive feedback loop. At the same time, the photothermal effect greatly enhanced the sensitivity of PDAC cells to chemotherapy, and finally, effectively inhibited tumor growth through the combination of PTT and chemotherapy. In another study of PTT in combination with chemotherapy, Zhan et al. designed a polymer micelle that was both ROS- and temperature-sensitive [118]. The excess ROS in TME can trigger a surface charge change in micelles, thereby releasing the outer indocyanine green (ICG). After ICG release, the micelles become smaller and penetrate more deeply into the tumor. At the same time, ICG can produce a photothermal effect under 808 nm laser irradiation, triggering the polymer to change from hydrophobic to hydrophilic, and releasing the loaded camptothecin into deep tumors to realize chemotherapy. In vitro and in vivo experiments showed that the combined treatment could effectively combat PDAC and improve the therapeutic effect. Semiconductor polymer NPs (SPN) composed of completely organic compounds have become photothermal agents for cancer treatment [119,120]. Compared with other inorganic nanomaterials, SPNs have unique characteristics such as better biocompatibility, and higher photothermal conversion efficiency and controllability. The utilization of SPNs for PTT has been well explored, but there are few reports on combination therapy. In their study, Shi et al. reported a ^177^Lu-labeled glucose-dependent insulinotropic polypeptide-targeting SPN for combined radiotherapy and PTT of PDAC [120]. As a result, these combined treatment techniques complemented each other and provided superior results to PTT or radiotherapy alone. This is a simple and effective way to fabricate SPN-based multifunctional diagnostic and therapeutic nanosystems. In addition to these widely studied materials, many novel nanomaterials have also been developed for PDAC PTT, such as single-walled CNTs [121] and graphene QDs [122], which exhibit excellent photothermal conversion efficiency and stability.

**Figure 4:**
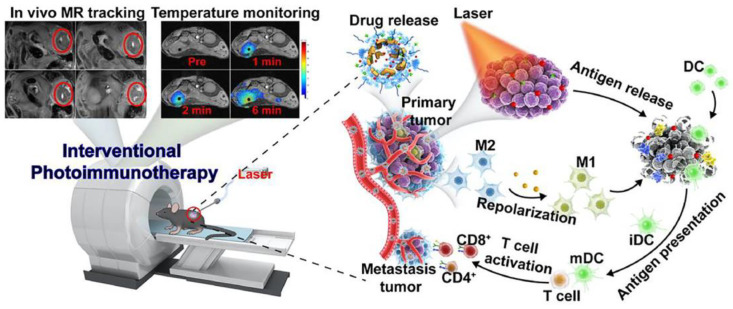
Schematic representation of IMQ@IONs/ ICG-mediated photothermal immunotherapy and real-time temperature monitoring for PDAC. Reprinted with permission from [124]. Copyright 2022, Elsevier.

Although PTT by itself is only suitable for local cancer treatment, combinations of PTT with other therapies have shown the benefits of inducing distant effects, in which local treatment suppressed not only the treated tumor but also the distal metastatic tumor [123]. Polydopamine (DP) is a kind of bionic coating material with excellent biocompatibility and photothermal conversion ability. In order to induce powerful anti-primary and metastatic tumor effects, Sun et al. designed a DP-coated ultra-small nanocarrier for the delivery of GEM and IDO inhibitors [123]. The nanosystem can not only inhibit tumor growth through the combination of chemotherapy and immunotherapy, but also further enhance the inhibitory effect on primary and metastatic tumors under the effect of PTT. Various nanosystems have been developed for phototherapy combined with immunotherapy. Nanomaterials can enhance the stability of photothermal agents or immune adjuvants, reduce side effects, and enhance therapeutic effect. In addition, these nanomaterials not only possess excellent photothermal properties, but can also induce remodeling of the tumor immune microenvironment. For example, Wang et al. constructed a nanosystem based on iron oxide loaded with imiquimod as an immune adjuvant and ICG as a photothermal agent (IMQ@IONs/ICG) [124]. Tumor antigen release was induced by PTT to stimulate DC cell activation and maturation. Moreover, IMQ@IONs/ICG could induce the polarization of M2-TAM to M1-TAM (Figure 4). This strategy ultimately eliminated the primary tumor and induced a robust immune response against metastasis and recurrence. Additionally, the multifunctional nanosystem also features MR imaging guidance and real-time temperature monitoring. It can accurately monitor the temperature change in tumor tissue during treatment. In another experiment, Li et al. assembled immunoregulatory thymopentin and ICG into nanofibrils for local combined PTT for PDAC [125]. The high aspect ratio of fibrillary nanostructures showed more pronounced retention in tumor tissue than that of other nanostructures. Their results showed that the combination of rapid PTT of tumor tissue and moderate systemic immune modulation, after a single injection and a single irradiation treatment, effectively suppressed tumor growth and tumor metastasis and minimized systemic side effects. PTT not only induced the anti-tumor immune response through ICD, but some studies have also shown that local hyperthermia can reduce the amount of CAF in PDAC, alleviate stromal disorders, and enhance the effect of chemotherapy [126]. Teng et al. reported an Abraxane-loaded MoSe_2_ nanosheet for chemotherapy combined with PTT [126]. Importantly, a reduction in the amount of CAF in response to the photothermal effects was confirmed via immunofluorescence analysis of α-SMA and vimentin. This therapeutic effect may provide a new perspective on PDAC treatment.

Although photothermal agents have led to progress in hyperthermia ablation, triggering drug release, and immune activation, their potential applications are still limited. Most photothermal agents are absorbed in the near-infrared (NIR) I region (600–1000 nm), which has limited light penetration depth. In contrast, NIR-II (1000–1700 nm) light is able to penetrate to deeper tissues and reduce light scattering, which is more suitable for PTT in deep tissues [127,128]. PTT in the NIR-II region can not only achieve high therapeutic effects and deep treatment, but can also minimize side effects. Geng et al. reported a platelet membrane-coated NIR-II nanosystem for tumor-targeted photothermal therapy [129]. The coating of the platelet membrane improved the hydrophilicity and targeting ability of NIR-II dye IR1048, which has excellent tumor suppressor effects in a variety of tumor models, including PDAC. The results showed that the nanosystem exhibited excellent photoacoustic imaging capability and significantly improved the photothermal conversion efficiency in the NIR-II window.

Overall, PTT has a strong and stable therapeutic effect in preclinical cancer studies. Current research focuses on designing targeted or stimulus-responsive nanosystems to improve the tumor specificity of photothermal agents, providing better results and fewer side effects. Especially when PTT is combined with chemotherapy or immunotherapy, it can produce a significant synergistic therapeutic effect. Based on the safety and efficacy of PTT, it has great translational potential in the treatment of PDAC.

**Table 5:** Summary of recent advances in well-designed nanosystem-mediated PTT, PDT, SDT, and CDT for the treatment of PDAC.

	Agents	Nanosystem	Treatment Strategies	Animal Model	Ref
**PTT**	Au nanoshell	Tf-GNRS-GEM	PTT + GEM	MIA PaCa-2 subcutaneous model	[117]
	Graphene	Graphene@Gold Nanostar/Lipid	PTT + gene therapy	Capan-1 subcutaneous model	[130]
	MoSe_2_	Abraxane@MoSe_2_	PTT reduced CAFs + Abraxane	PDX model	[126]
	ICG	CPT@PAAB@ICG	PTT + chemotherapy	BXPC3 subcutaneous model	[118]
	SPN	^177^Lu-SPN-GIP	PTT + RT + SPECT/CT	CFPAC-1 subcutaneous model	[120]
	SWNT	IGF-1R-targeted SWNT	PTT	BXPC-3 orthotopic model	[121]
	ICG	IMQ@IONs/ICG	IPTT + immunotherapy + MRI	Panc02-H7 orthotopic model	[124]
	Conjugated small molecule	DCTBT-loaded liposomes	PTT + PDT + FLI	PANC-1 orthotopic model	[131]
	Dopamine	NLG/PGEM/dp NPs	PTT + GEM + immunotherapy	Panc02 subcutaneous model	[123]
**PDT**	SiPc	PFC/SiPc@PS@PNIPAM-Au980-DOX	PDT + PTT + chemotherapy	MIA PaCa-2 orthotopic model	[132]
	PPIX	Perfluoropentane-doped oxygen MBs	PDT + O_2_	Panc02 orthotopic model	[133]
	DiD	PF_11_DG	PDT + O_2_ + GEM + immunotherapy	Panc02 subcutaneous model	[134]
	RB, Ce6	UCNP/RB, Ce6	Dual PDT	PANC-1 subcutaneous model	[135]
	PPa	Supramolecular prodrug nanosystem	PDT + immunotherapy	Panc02 subcutaneous model	[136]
	Ce6	Ce6-GVS NPs	PDT + chemotherapy + pro-apoptotic signal	PANC-1 subcutaneous model	[137]
	TBD-3C	Membrane-anchoring photosensitizer	PDT + pyroptosis + immunotherapy	KPC orthotopic model	[138]
**SDT**	TiO_2_	Tablet-like TiO_2_/C nanocomposites	SDT	Panc02 subcutaneous model	[139]
	IR780	IR780@O2-FHMON	SDT + O_2_	PANC-1 subcutaneous model	[140]
	TCPP	Ti-TCPP MOF	SDT	BxPC-3 orthotopic model	[141]
	PPIX	PMPS NDs	SDT + immunotherapy	Panc02 orthotopic model	[142]
	IR780	Pt@ZIF-90@Gem@IR780	SDT + chemotherapy	BxPC-3 subcutaneous model	[143]
**CDT**	Cu	HAS-MnO_2_-CuS	CDT + PTT	Panc02 subcutaneous model	[144]
	Fe^2+^	HFePQS	CDT + immunotherapy	KPC orthotopic model	[81]
	HPPH	HMON-Au-Col@Cu-TA-PVP	CDT + PDT	BxPC-3 subcutaneous model	[145]
	AIPH	AIPH@Cu-MOF	CDT + SDT	Panc02 orthotopic model	[146]

### 2.6. Enhanced Photodynamic Therapy (PDT) Based on Tailored Nanosystems

**Figure 5:**
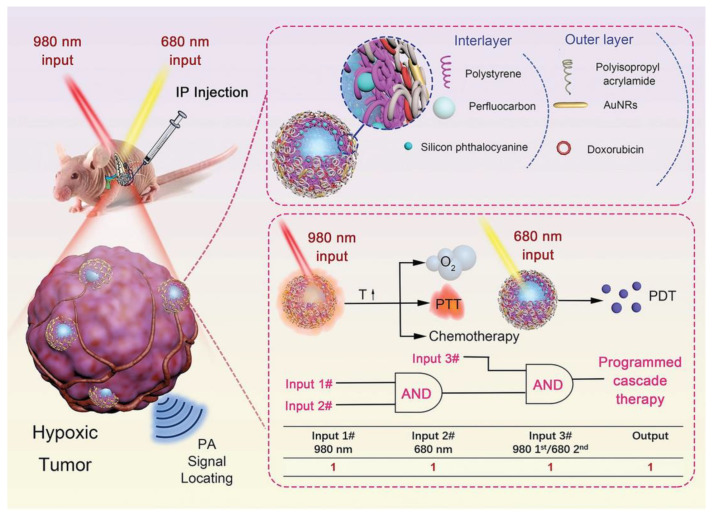
Schematic diagram of PSPP-Au980-D-mediated controllable release of “oxygen bomb” and programmed cascade therapy process. Reprinted with permission from [132]. Copyright 2022, John Wiley and Sons.

PDT is a minimally invasive treatment that involves shining a laser light on a photosensitizer (PS) that transfers absorbed photon energy to the surrounding oxygen, resulting in the generation of ROS primarily consisting of singlet oxygen (^1^O_2_) [147]. The ROS produced can trigger oxidative damage to mitochondria, leading to apoptosis [148]. PDT has shown advantages in existing clinical trials and has emerged as a potential alternative therapy for patients with advanced PDAC [149,150]. However, there are still some shortcomings in the clinical application of PDT, including poor water solubility of PS and easy aggregation, tumor hypoxia, and limited efficacy in invasive tumors [147]. In preclinical studies, nanosystem-mediated PDT has been shown to overcome these deficiencies through the design of various corresponding strategies (Table 5). Hypoxia at the tumor site and the non-specific accumulation of PS are the key factors limiting the efficacy of PDT. Based on this, Xu et al. developed a PS- and oxygen-targeted delivery system to alleviate tumor hypoxia, reduce the non-specific accumulation of PS, and enhance the efficacy of PDT [133]. They prepared a perfluoropentane-doped oxygen microbubble (OPMB) for efficient oxygen delivery and an ultra-small PEG-modified protoporphyrin IX micelle (PPM) for PDT treatment. Protoporphyrin IX is a heme prerequisite with excellent photosensitive activity, but its hydrophobicity and non-target tissue accumulation limit its application [133]. Conjugated PEG and protoporphyrin IX self-assembled into ultra-small PPMs, which resolved the hydrophobicity of protoporphyrin IX and reduced nonspecific accumulation. Furthermore, the disruption of OPMB with ultrasound resulted in an approximately 2.2-fold increase in tumor-specific PPM accumulation. In addition, tumor oxygenation was increased from 22% to 50% by the MB disruption of oxygen release, which not only increased ^1^O_2_ production but also significantly decreased the expression of HIF-1α, thus preventing angiogenesis and epithelial–mesenchymal transition. This strategy has been shown to significantly prolong the survival of mice with orthotopic PDAC. In another study, Zhang et al. designed a cascade therapy strategy tailored for hypoxic orthotopic PDAC [132]. They prepared a nanosystem called PSPP-Au_980_-D, which was composed of a bilayer functionalized polymer encapsulating perfluorocarbon (PFC). In their design, the first step is to irradiate the nanosystem with a 980 nm laser to induce photothermal effects, triggering the explosive release of oxygen and doxorubicin. The second step is to use a 680 nm laser to trigger PDT under high oxygen content. The controlled release of the “oxygen bomb” and the programmed cascade therapy strategy provided a new way to suppress hypoxic tumors (Figure 5). In addition to improving the efficacy of PDT by increasing the oxygen content, highly cytotoxic free radicals are produced via the type I pathway and oxygen dependence is significantly reduced. Therefore, type I PDT is capable of producing highly potent toxic free radicals under hypoxia. However, the development of type I PS with long-wavelength absorption regions for deep tissue penetration remains a great challenge. Based on this, Li et al. prepared a PS with aggregation-induced emission (AIE) properties, namely DCTBT [131]. Through clever molecular engineering, DCTBT has powerful AIE properties, NIR-II absorption, and type I PDT and PTT function. DCTBT was encapsulated by liposomes modified with targeted peptides, and then, delivered to the tumor site. The results showed that DCTBT-loaded liposomes exhibited significant tumor growth inhibition in a PDAC mouse model following NIR-II fluorescence imaging-guided synergistic treatment of type I PDT and PTT. The NIR-IIb region (1500 to 1700 nm) has recently been identified as a promising region for deeper tissue penetration [135]. Based on this, Pham et al. developed a dual PDT nanosystem with a 1550 nm light response to effectively promote ^1^O_2_ production [135]. Rose red (RB) and Ce6 were loaded into SiO_2_-coated core-shell upconverted NPs to form a nanosystem. This nanosystem can further activate RB and Ce6 by emitting green (~550 nm) and red (~670 nm) light under laser irradiation at 1550 nm. The simultaneous excitation of double PS produced a large amount of ^1^O_2_, thus producing a powerful PDT effect. In vitro and in vivo experiments demonstrated that this dual PDT nanosystem exhibited enhanced antitumor effects at a single dose of treatment compared to single PS. The combination of enhanced dual PDT and 1550 nm photoexcitation is expected to provide new avenues for PDAC treatment.

In addition to strategies that enhance the effects of PDT, such as alleviating hypoxia and double PS, synergistic combinations of multiple therapeutic strategies are expected to improve the dismal efficacy of PDAC (for example, the combination of PDT and chemotherapy or immunotherapy). Zhu et al. developed a PDAC treatment strategy that combines PDT, chemotherapy, and pro-apoptotic signals [137]. The nanosystem consists of Ce6 and a pro-apoptotic peptide-GEM conjugate. The pro-apoptotic peptide-GEM conjugate was linked by a ROS-sensitive linker, and then, self-assembled with Ce6 to form Ce6-GVS NPs. Upon irradiation using a 660 nm laser, the nanosystem generated ROS and triggered the release of GEM and pro-apoptotic peptides. The strong tumor-suppressive effect of the combined strategy was confirmed in vitro and in vivo. Sun et al. designed a supramolecular prodrug nanoplatform to promote PDAC photodynamic immunotherapy by regulating glucose metabolism [136]. The prodrug NPs were prepared via the host–guest interaction between β-cyclodextrin-grafted hydronic acid and a supramolecular prodrug nanosystem to deliver pyropheophorbide a (PPa) and a bromodomain and extraterminal protein 4 inhibitor (JQ1) for the photoimmunotherapy of PDAC. PPa can produce PDT effects under 671 nm laser irradiation, thereby activating the ICD effect and causing the activation and infiltration of CD8^+^T cells. JQ1 can inhibit PDT-enhanced glycolysis and down-regulate the expression of PD-L1 in tumor cells to inhibit PDT-induced immune tolerance. Therefore, this is the first study to demonstrate the role of supramolecular NPs in the regulation of tumor glycolytic and immune microenvironments and may be a novel strategy to promote photodynamic immunotherapy for PDAC. Hypoxia not only severely limits the efficacy of PDT, but also can regulate a variety of immune cells to promote the tumor phenotype and enhance the immunosuppressive function of MDSC and Treg, resulting in a significant immunosuppressive effect. Therefore, improving tumor hypoxia can effectively enhance the antitumor response mediated by PDT and immunomodulators. Wang et al. reported an oxygen delivery PFC carrier for increasing oxygen levels in tumors and enhancing photodynamic immunotherapy effects [134]. The nanosystem specifically accumulated and enhanced oxygen levels within the tumor site. Under laser irradiation, DiD produced a large number of ROS, induced the generation of the ICD effect, and triggered the responsive release of GEM; this effectively stimulated CD8^+^T cells and NK cells and inhibited MDSC, and finally produced a strong anti-tumor effect. Previous studies have shown that PDT can induce apoptosis, necrosis, autophagy, and ICD [151]. Recently, a novel membrane-targeted PS (TBD-3C) has been shown to activate cancer cell pyroptosis [152]. Wang et al. further evaluated the immune activation effect of TBD-3C-induced pyroptosis [138]. They found that PDT induced by TBD-3C could induce pyroptosis, activate antitumor immunity, and significantly inhibit the growth of PDAC. Their findings lay the foundation for photo-controlled antitumor immunotherapy induced by pyroptosis.

In summary, PDT is a powerful and effective treatment for cancer. The main obstacles to PDT treatment in PDAC are severe internal hypoxia and limited light penetration depth. A variety of nanosystem design strategies to alleviate hypoxia and enhance light penetration depth have been widely developed. Recent research aims to overcome the shortcomings of PDT treatment through multifunctional nanosystems and enhance the efficacy of PDT by combining multiple treatment modalities. Although the integration of multiple functions in one nanosystem is beneficial in overcoming the drawbacks of each treatment modality, the corresponding increase in the complexity of the manufacturing process makes clinical translation difficult. Future research may require more efforts to fabricate nanosystems that are beneficial in overcoming multiple dilemmas through simple and convenient synthetic routes.

### 2.7. Elaborate Nanosystems for PDAC Sonodynamic Therapy (SDT)

**Figure 6:**
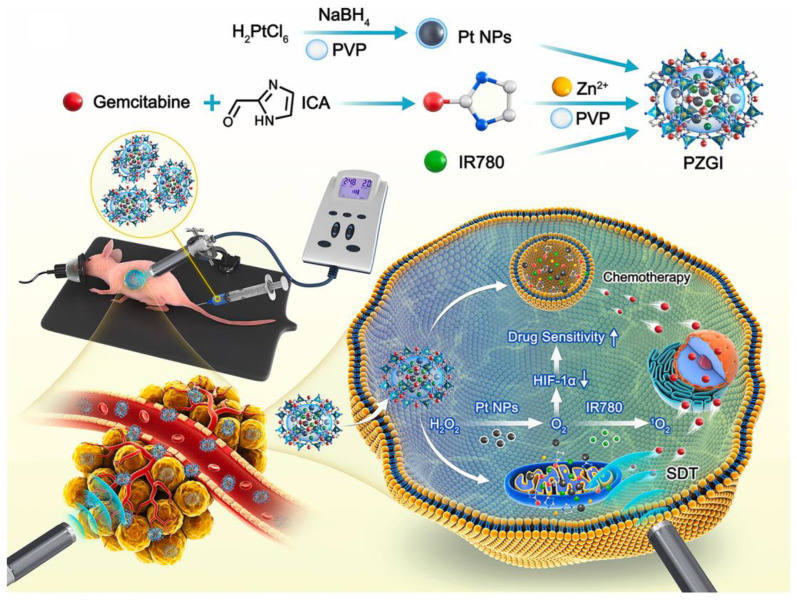
Schematic diagram the pH/ATP dual responsive Pt@ZIF-90@Gem@IR780 for synergistic chemo-sonodynamic therapy of PDAC. Reprinted with permission from [143]. Copyright 2021, Elsevier.

SDT, as a promising non-invasive treatment, has attracted more and more attention in recent years. SDT relies on low-intensity ultrasound to trigger the production of cytotoxic ROS by sonosensitizers, thereby inducing cell damage [153]. Compared with PDT, SDT has greater advantages in tissue penetration depth. Using the penetration of ultrasound into deep tissues, SDT has thus emerged as a promising alternative for the treatment of deep tumors while still maintaining its advantages over conventional cancer treatment modalities. Although preclinical studies of SDT have shown promising results in various tumor cells, its therapeutic efficacy of tumor elimination in vivo is still far from satisfactory [153]. Driven by advances in nanotechnology, many new nanosystems have been developed in this emerging field to enhance the therapeutic efficacy of SDT (Table 5). Conventional SDT still relies on the intratumoral supply of oxygen, which is required for ROS production. However, the utility of SDT in PDAC is limited due to inherent intratumoral hypoxia. Recent studies have shown that titanium dioxide (TiO_2_) has the characteristic of responding to ultrasound [139]. Ultrasound can induce TiO_2_ to mediate the production of cytotoxic free radicals and superoxide, and can achieve efficient oxygen-independent type I SDT. However, the single TiO_2_ material has some limitations related to its stability and energy transfer efficiency. To this end, Cao et al. prepared a TiO_2_/C nanocomposite for type I SDT-mediated PDAC treatment [139]. This novel TiO_2_/C nanocomposite has excellent stability and can effectively produce ROS even under hypoxic conditions. Another key determinant of the efficacy of SDT is the subcellular localization of the sonosensitizer, as most ROS generated have a very short lifetime and limited diffusion length [154]. Recent evidence suggests that sonosensitizers located near DNA are able to induce more intense oxidative damage, thereby achieving a powerful therapeutic effect [155]. Zhang et al. reported a nuclear-targeted Ti-tetrakis(4-carboxyphenyl)porphyrin (TCPP) MOF that can exert type I SDT efficacy as a sonosensitizer [141]. This novel MOF can effectively target the nucleus and directly break DNA through the SDT effect. In addition, the MOF can efficiently produce ROS in hypoxic environments, and thus, has great prospects for the treatment of hypoxic tumors. In another study, Chen et al. designed an ER-targeted nanodroplet that selectively accumulated in the ER, generated large amounts of ROS, induced intense ER stress, and finally, triggered strong ICD effects [142]. In addition to enhancing the ability to produce ROS under hypoxic conditions through type I SDT, another strategy is to enhance the efficacy of type II SDT by providing oxygen. Chen et al. constructed an oxygen-generated nanoplatform mediated by modified fluorocarbon (FC) chains for efficient hypoxic SDT treatment [140]. The nanoplatform consisted of MSNPs functionalized with FC chains loaded with the sonosensitizer IR780. This oxygen delivery protocol effectively alleviated the intratumor hypoxia state of PDAC and produced sufficient ROS to kill tumor cells under the induction of ultrasound. In another study, Chen et al. designed a pH/ATP dual responsive nanoplatform based on zeolitic imidazolate framework-90 to alleviate PDAC hypoxia and enhance SDT efficacy [143]. In this nanosystem (Pt@ZIF-90@Gem@IR780), platinum (Pt) NPs were added to exert catalase activity and promote the conversion of hydrogen peroxide into oxygen in the tumor to increase the oxygen content. IR780 and GEM were co-loaded onto the nanoplatform as SDT therapeutic agents and chemotherapy agents. After catalysis by Pt NPs, the hypoxia situation in the tumor was relieved, ultimately leading to an enhanced SDT effect and chemotherapy sensitivity (Figure 6). Ultrasound-targeted MB destruction is an emerging technology in the field of drug delivery that involves the use of low-intensity ultrasound to destroy MBs at the target site to release loaded cargo or encapsulated gas [156]. When oxygen and sonosensitizer are loaded into MBs, under the stimulation of ultrasound, on the one hand, the cavitation effect of MBs is used to rupture the MBs and release oxygen; on the other hand, the sonosensitizer can produce a large amount of ROS efficiently under the promotion of oxygen. Nesbitt et al. combined oxygen MB-GEM with RB-functionalized oxygen MB for chemo-sonodynamic therapy, which effectively inhibited tumor growth and resulted in the decreased expression of markers related to PDAC progression, demonstrating great potential for the treatment of PDAC [157]. In another study, Sheng et al. added magnetic NPs into MB shells to deliver MBs in a targeted manner under the action of an external magnetic field [158]. They constructed oxygenated magnetic MBs with 5-FU and RB attached to the surface for PDAC-targeted therapy. The combination of an external magnetic field and ultrasound resulted in a 48.3% reduction in tumor volume in orthotopic PDAC compared with controls. Their results demonstrated the effectiveness of using a magnetic targeting MB platform for the highly targeted therapy of PDAC.

To summarize, SDT is a new treatment that has been developed for PDAC in recent years, and there are few related studies. However, SDT has great potential for the treatment of PDAC due to its multiple advantages, especially its deep penetration ability. Future studies should focus more on the enrichment of sonosensitizer at the tumor site, subcellular localization, tumor hypoxia, and other issues to achieve a more powerful therapeutic effect of SDT.

### 2.8. Custom-Made Nanosystems for Enhanced Chemodynamic Therapy (CDT)

Another type of treatment that damages cells by producing hydroxyl radicals (·OH) is called CDT [159] (Table 5). Different from PDT or SDT, CDT does not depend on light or ultrasound, but on the Fenton reaction chemical process of endogenous hydrogen peroxide (H_2_O_2_) to OH [159]. Therefore, CDT is an effective therapeutic strategy to overcome tissue depth and the hypoxic microenvironment. However, the insufficient H_2_O_2_ content in the microenvironment will weaken the therapeutic effect of CDT [144]. Therefore, increasing the in situ production of H_2_O_2_, and thereby enhancing Fenton or Fenton-like reaction, is a promising strategy to enhance CDT efficacy. Ultra-small AuNPs are a kind of nanoenzyme with glucose oxidase activity, and can catalyze the formation of H_2_O_2_ from glucose [160]. Using this property, Li et al. designed a nanosystem based on hollow mesoporous organosilica nanoparticles (HMONs) for PDT- and CDT-mediated PDAC treatment [145]. The photosensitizer HPPH was hybridized to the HMON skeleton through in situ growth. Ultra-small AuNPs were immobilized in the HMON cavity via thiol groups. Finally, Cu^2+^-chelated tannic acid was deposited on the surface of the HMONs. The nanosystem first catalyzed glucose to produce H_2_O_2_, which promoted the occurrence of Fenton-like reactions and induced strong CDT therapeutic effects. In addition, under NIR irradiation, the resulting PDT effect further synergistically killed tumor cells. This well-designed therapeutic cascade effectively enhanced ROS-mediated anti-PDAC therapy. Recent studies have found that hyperthermia produced by PTT can promote the catalytic efficiency of the Fenton reaction and contribute to the realization of efficient synergistic cancer therapy [161]. Based on this, Sun et al. developed a photothermal Fenton nanocatalyst for PDAC synergistic PTT and CDT [144]. The nanosystem was synthesized by combining copper sulfide (CuS) with manganese dioxide coated with human serum albumin. CuS acted as a nanocatalyst to produce toxic ·OH in a Fenton-like reaction with H_2_O_2_. Furthermore, NIR-II irradiation not only ablated tumor cells via hyperthermia, but also accelerated the Fenton-like response. The results showed that the synergistic treatment of PTT and CDT completely inhibited tumor growth in Panc02 tumor-bearing mice. In addition to the combination of PTT, Sun et al. designed a Cu-MOF for SDT and CDT, which was able to efficiently induce cellular free radical production under hypoxic conditions [146]. On the one hand, this Cu-MOF will degrade under hypoxic conditions, releasing Cu^2+^ and sonosensitizer. When exposed to ultrasound, the sonosensitizer produced nitrogen bubbles and alkyl radicals in an oxygen-independent manner. On the other hand, the released Cu^2+^ consumed a high level of GSH in the tumor and transited into Cu^+^, which then underwent a Fenton-like reaction with H_2_O_2_ to produce ·OH. This hypoxic TME responsive sono-chemodynamic therapy strategy provided an effective way to achieve oxygen-independent free radical generation.

It was found that Fe(III)/Fe(II) could induce polarization of M2-TAM to the M1-TAM phenotype [162]. Because of the stimulative role of M2-TAM in PDAC stromal formation, Fe(III)/Fe(II) is expected to comprehensively improve PDAC therapy by simultaneously killing tumor cells via CDT and reprogramming the PDAC stromal microenvironment. To test this hypothesis, Chen et al. constructed a nanosystem to simultaneously kill tumor cells and reshape the matrix [81]. Fe^2+^ and a strong acid environment are essential for the Fenton reaction to occur. Therefore, they introduced SO_2_ into the nanosystem as a reducing agent to convert Fe^3+^ to Fe^2+^ and produce sulfuric acid. In addition, GSH-responsive groups were introduced into the nanosystem to achieve controllability. The excess GSH in tumor cells triggered the rapid release of Fe^3+^ and SO_2_, which further promoted the conversion of Fe^3+^ to Fe^2+^ and the production of sulfuric acid. Finally, the Fenton reaction produced a large amount of ·OH to kill tumor cells. However, in TAM cells, the Fenton response was not triggered due to insufficient GSH. Instead, Fe^3+^ innocuously induced TAM to polarize to the M1 phenotype, ultimately downregulating pro-fiber signaling and alleviating stromal disorders. Thus, this carefully tailored nanosystem exploited the unique redox homeostasis within the TME for different therapeutic purposes.

Overall, CDT is a promising treatment modality for PDAC, and its potent free radical production capacity can exert potent cytotoxic effects. In addition, it is independent of external energy and is not limited by penetration depth, which also helps to overcome the difficulties of PDAC treatment. However, CDT also has its associated limitations, such as insufficient H_2_O_2_ levels, highly acidic environments, and the catalytic efficiency of Fenton reactions. The design of more sophisticated nanosystems is needed to fully consider the limitations of CDT and make up for its shortcomings.

## 3. Conclusions and Challenges

PDAC is a malignant tumor with an extremely poor prognosis, and its survival prognosis has not improved significantly over the years. The main reason is that the existence of various dilemmas and obstacles makes the disease resistant to a variety of treatment methods. The resistance to and side effects of chemotherapy drugs have greatly limited their efficacy in the treatment of PDAC. Researchers have attempted to improve outcomes by targeting specific pathways, but have experienced many failures. In addition, initial clinical attempts to target tumor stroma have failed. Even immunotherapies that have been successful in a variety of human tumors have been unable to break down the barriers to PDAC treatment. The development of nanotechnology brings new hope for the treatment of PDAC. Well-designed smart nanosystems have been extensively developed to overcome various barriers in PDAC therapy, such as charge-reversal nanosystems, size-transform nanosystems, cascade-targeted nanosystems, and cluster bomb nanosystems [80]. These nanosystems are endowed with a variety of powerful capabilities that can target therapeutic needs, and have shown encouraging results in preclinical trials.

However, only a small number of nanomaterials have been approved for clinical use and they are focused on relatively simple products such as liposomes. Therefore, the clinical translation of nanomedicine is one of the major challenges for researchers designing nanosystems. Many factors hinder the clinical translation of nanomaterials, including the stability of nanomaterials, effective biodistribution, in vivo toxicity, and biodegradation mechanisms [21]. For example, Vadim et al. reported that tiny rare-earth fluoride NPs carry the risk of promoting tumor cell growth through electrical polar interactions [163]. Other nanomaterials such as gold NPs, MSNPs, and ceria NPs have been reported to have tumor-promoting properties [164,165,166]. It is estimated that about 20% of nanomaterials in clinical trials fail due to safety issues. Therefore, the toxicity of nanomaterials is a non-negligible problem, so the safety of nanomaterials must be guaranteed before entering into clinical transformation. The development of toxicological analyses of nanomaterials has helped to identify key physicochemical characteristics that contribute to their toxicity and to design safer strategies [167]. By optimizing the physicochemical properties of nanomaterials, the toxicity of nanomaterials can be minimized while their biological efficacy is maximized. In addition, issues related to raw material costs, manufacturing costs, and standardized mass production are also factors to be considered. Designing ever more complex nanosystems will not enable us to escape this transformation mess. To escape this predicament, we must critically rethink the current and future directions of nanomedicine. In addition to the development of intelligence and versatility, more attention should be paid to the practical transformation value of nanosystems. For example, in the process of designing and manufacturing nanomaterials, the physical and chemical characterization of nanomaterials should be fully completed to ensure the stability and biological safety of the materials [168].

The transport of nanosystems in PDAC tumor tissues is another challenge limiting their effectiveness. Most nanomedicines that are currently in clinical trials rely on EPR effects. However, the stromal barrier of PDAC severely limits the EPR effect. Recent studies have found that transcytosis is an effective targeting strategy that can complement the EPR effects of PDAC drug delivery [169]. Related studies include the discovery of vesico-vacuolar organelle-like structures in PDAC [170,171], the iRGD peptide-triggered mass transcytosis pathway [172], caveolae-mediated transcytosis mediated by albumin-based nanosystems [173,174], urokinase plasminogen activator receptor-mediated transcytosis [175], and macropinocytosis-mediated transcytosis activated by cationic NPs [79]. These studies suggest that the active transport mode of transcytosis can bypass the barrier of passive diffusion and enable the active penetration of nanomaterials into tumors. Future research designs should take into account the lack of EPR effects, as well as the advantages of active transport.

On the other hand, anti-stroma therapy strategies developed in preclinical studies have shown enhanced efficacy, but many of these strategies have resulted in clinical failure [6]. These results have sparked debate about the pro-tumor and anti-tumor effects of the stroma in PDAC. Our understanding of the biological mechanisms of the PDAC stroma is relatively preliminary compared to other PDAC-related mechanisms [6]. In fact, the PDAC stroma is a heterogeneous equilibrium system, and disruption of this equilibrium may have harmful consequences, such as promoting tumor invasion and metastasis [176,177]. The two-sided nature of the PDAC stroma should be fully considered in the design of nanosystems, and the stroma regulation strategy should be carefully evaluated and formulated.

Current immunotherapy strategies tend to use combined regulatory approaches [176]. However, the immune microenvironment of PDAC is extremely complex and variable. There are still significant limits to our understanding and regulation of the immune microenvironment. It is important to recognize that patients with PDAC may have more than one immune deficiency, and what is even more challenging is that immune deficiency may change during the treatment [178]. Therefore, choosing the right combination of treatments at the right point in time is critical.

Additionally, nanomaterials face the challenge of recognition and clearance by the immune system. The successful use of Abraxane^®^ (nab-paclitaxel) illustrates the advantages of endogenous proteins as drug carriers [179]. Cell-derived exosomes are also being used in clinical trials for PDAC diagnosis (NCT03032913). The evidence suggests that biomimetic nanomaterials may have greater potential.

Finally, given the complexity of the TME of PDAC, the selection of tumor models in preclinical studies is of great importance. Subcutaneous xenograft models may struggle to mimic the stromal microenvironment of PDAC, even when tumor cells are co-injected with PSCs. The genetically engineered mouse model can better summarize the molecular biological and histopathological features of human PDAC, including similar precursor lesions, the dense fibrotic matrix, and the immunosuppressive microenvironment [180]. In addition, the PDX model has high fidelity in PDAC modeling and is a powerful platform for both basic and translational research [181]. However, due to its high cost and long duration, the PDX model is not an ideal platform for large-scale research. Patient tumor-derived organoid models that retain the genetic and phenotypic characteristics of PDAC are rapidly developing as an effective method for mimicking patient disease in vitro [181].

Collectively, a series of well-designed smart nanosystems hold infinite promise for the treatment of PDAC. However, there is still a long way to go to overcome the dilemma of PDAC treatment and realize clinical transformation. With the joint efforts of researchers from multiple disciplines, nanotechnology is expected to achieve new breakthroughs for PDAC treatment.

## Figures and Tables

**Table 1:** Clinical trials of nanosystem therapeutic strategies for PDAC (obtained from https://clinicaltrials.gov/, accessed on 1 October 2022).

Nanosystems	Nanomaterials	Study Design	Identifier
Nano-SMART: AGuIX gadolinium-based NPs with radiotherapy	NPs	Phase I/II trial	NCT04789486
Hafnium oxide-containing NPs NBTXR3 with radiation therapy	NPs	Phase I study	NCT04484909
AA NABPLAGEM: ascorbic acid, NP paclitaxel protein-bound, cisplatin, and GEM	NPs	Phase Ib/II trial	NCT03410030
SNB-101: nano-particle formulation of SN-38, the active metabolite of irinotecan (CPT-11)	NPs	Phase I study	NCT04640480
IMX-110: NPs encapsulating a Stat3/NF-kB/poly-tyrosine kinase inhibitor and low-dose doxorubicin	NPs	Phase I/IIa trial	NCT03382340
BIND-014: docetaxel NPs for injectable suspension	NPs	Phase I study	NCT01300533
CALAA-01: a targeted nanocomplex that contains anti-R2 siRNA	NPs	Phase I study	NCT00689065
CYT-6091: a PEGylated colloidal gold-rhTNF nanomedicine	Colloidal AuNPs	Phase I study	NCT00356980
Paclitaxel liposome and S-1	Liposome	Single-arm, prospective study	NCT04217096
Mitoxantrone hydrochloride liposome	Liposome	Phase II study	NCT05100329
Docetaxel and liposomal doxorubicin chemotherapy with enoxaparin	Liposome	Phase II trial	NCT00426127
PanDox: thermosensitive liposomal doxorubicin	Liposome	Phase I study	NCT04852367
Aroplatin (liposomal NDDP, L-NDDP) and GEM	Liposome	Phase I/II study	NCT00081549
MM-398 (nanoliposomal irinotecan, Nal-IRI) and Ferumoxytol	Liposome	Phase I study	NCT01770353
Genexol-PM (cremophor EL-free polymeric micelle of paclitaxel) and GEM	Micelle	Phase I/II trial	NCT00882973
NC-6004 (a novel micellar cisplatin formulation) and GEM	Micelle	Phase III study	NCT02043288

**Table 3:** Recent advances in nanosystem-mediated gene therapy for PDAC.

Gene	Gene Function	Gene Therapeutics	Nanocarrier	Animal Model	Ref
*KRAS^G12D^*	Initiation, development, and metastasis of the tumor	Peptide nucleic acid	LDH	PANC-1 subcutaneous model	[56]
*HIF1α*	Regulates tumor invasion, proliferation, angiogenesis, and drug resistance	*HIF1α* siRNA	Lipid–polymer hybrid NPs	PANC-1 subcutaneous and orthotopic models	[57]
*KRAS* and *P53*	Drug resistance-related	Cas9-ribonucleoproteins and adenine-base editors	Antibody-conjugated nanoliposomal particles	PANC-1 subcutaneous model	[58]
MicroRNA-21 (*miR-21*)	Oncogenic activity, cancer initiation and progression	Antisense oligonucleotide-*miR-21* (ASO-*miR-21*)	Polyethylene glycol-polyethyleneimine-magnetic iron oxide NPs	MIA PaCa-2 subcutaneous model	[59]
*miR-21*	Modulation of apoptosis, Akt phosphorylation, and invasive behavior	*miR-21* inhibitor	Dendrimer-entrapped gold NPs	SW1990 subcutaneous model	[60]
*KRAS*	Tumor growth and proliferation	*KRAS* siRNA	Hydroxyapatite NPs	PANC-1, CFPAC-1, and BXPC-3 cells	[61]
*TRAIL* gene	Induces apoptosis in tumor cells	*TRAIL* pDNA	Branched polyethyleneimine	BxPC3 orthotopic model	[62]
*Relaxin* (*RLN*) gene	Decreases fibrosis	*RLN* pDNA	Lipid–protamine–DNA NPs	Allografting KPC model	[63]
Methyl-CpG-binding domain 1 (*MBD1*)	Epigenetic regulation and transcriptional repression	*MBD1* siRNA	Multi-walled CNTs	BxPC-3 subcutaneous model	[64]
*miR-634*	Mitochondrial homeostasis, antiapoptosis signaling, redox, and autophagy–lysosomal degradation	Ds-*miR-634* mimics	Lipid NPs	BxPC-3 subcutaneous model	[65]
*Bcl2*	Anti-apoptotic	*Bcl2* siRNA	Lipid–calcium–phosphate NPs	KPC orthotopic model	[66]
*TRAIL*	Induces apoptosis in tumor cells	*TRAIL* pDNA	ROS-responsive polymeric nanocarriers	BxPC-3 orthotopic model	[67]
*miR-519c*	Binds to hypoxia-inducible factor-1α (HIF-1α) mRNA, and can inhibit HIF-1α expression	*miR-519c*	Redox-sensitive polymeric micelles	MIA PaCa-2R orthotopic model	[68]
Nerve growth factor (*NGF*)	Promotes the growth of neurites and stimulate neurogenesis	*NGF* siRNA	Gold nanoclusters	PANC-1 subcutaneous, orthotopic, and PDX model	[69]
Histone deacetylase 1 (*HDAC1*) and *KRAS*	Regulates cell transformation, survival, invasion, and metastasis	siRNA	PEGylated GO nanosheets	MIA PaCa-2 subcutaneous model	[70]

**Table 4:** Recent advances in nanosystem-mediated modulation of PDAC stroma.

Target	Strategy	Nanocarrier	Animal Model	Ref
PSCs	Calcipotriol	Self-assembled prodrug NPs	PSCs/AsPC-1 co-implanted orthotopic model	[73]
PSCs	Nitric oxide (NO)	NO donor S-nitroso-N-acetylpenicillamine (SNAP)-loaded liposomes	PANC-1 and PSC subcutaneous and orthotopic model	[74]
CAFs	CAF-responsive	Thermosensitive liposomes	PAN02/NIH3T3 subcutaneous model	[75]
CAFs	α-Mangostin (α-M)	CREKA peptide-modified PEG–PLA nanoplatform	PANC-1/NIH3T3 subcutaneous model	[76]
TGF-β	Vactosertib (VAC)	Paclitaxel nanosphere-loaded VAC liposomes	PANC-1 orthotopic model	[77]
TGF-β	LY2109761 (a novel TGF-β receptor type I kinase inhibitor)	Alternating copolymer	PANC-1 orthotopic model	[78]
Endothelial cells	Transcytosis	Polymer–drug conjugate	BxPC-3 orthotropic tumor model	[79]
Endothelial cells	Transcytosis	Dendrimer–drug conjugate	BxPC-3 subcutaneous, orthotropic, and PDX subcutaneous tumor models	[80]
Tumor-associated macrophages (TAMs)	Fe(III)/Fe(II)	Tailored nanocomplex	KPC1199 orthotopic model	[81]
ECM	Collagenase	Collagenase-encapsulated liposomes	KPC orthotopic model	[82]
ECM	Collagenase	Collagenase-loaded hollow TiO2 NPs	PDX orthotopic model	[83]

## Data Availability

Not applicable.

## Abbreviations

AIE: aggregation-induced emission; APCs: antigen-presenting cells; CAFs: cancer-associated fibroblasts; CDT: chemodynamic therapy; Ce6: chlorin e6; CNTs: carbon nanotubes; CRT: calreticulin; CTL: cytotoxic T cell; CuS: copper sulfide; DCs: dendritic cells; dCTP: deoxycytidine triphosphate; ECM: extracellular matrix; ER: endoplasmic reticulum; 5-FU: 5-fluorouracil; GEM: gemcitabine; GEM-HSA-NP: GEM-loaded human serum albumin nanoparticle; GGT: γ-glutamyl transpeptidase; GO: graphene oxide; GSH: glutathione; hENT1: human equilibrative nucleoside transporter 1; hNTs: human nucleoside transporters; H_2_O_2_: hydrogen peroxide; ICD: immunogenic cell death; ICIs: immune checkpoint inhibitors; KPC: *KRAS*-induced pancreatic cancer; LDH: layered double hydroxides; MDSCs: myeloid-derived suppressor cells; miRNA: microRNA; MMP-2: matrix metalloproteinase 2; MOF: metal–organic framework; MSNPs: mesoporous silica NPs; NO: nitric oxide; NPs: nanoparticles; ·OH: hydroxyl radicals; PanIN: pancreatic intraepithelial neoplasia; PDAC: pancreatic ductal adenocarcinoma; pDNA: plasmid DNA; PDT: photodynamic therapy; PDX: patient-derived xenograft; PFC: perfluorocarbon; PS: photosensitizer; PSC: pancreatic stellate cell; PTT: photothermal therapy; QDs: quantum dots; RB: rose red; SDT: sonodynamic therapy; siRNA: short interfering RNA; α-SMA: α smooth muscle actin; SNAP: S-nitroso-N-acetylpenicillamine; SPIO: superparamagnetic iron oxide; TAMs: tumor-associated macrophages; Tf: transferrin; TGF-β: transforming growth factor-β; TLR: Toll-like receptor; Tregs: regulatory T cells; VAC: vactosertib.
